# O Impacto da Educação na Mortalidade por Todas as Causas após Infarto do Miocárdio com Supradesnivelamento do Segmento ST (IAMCSST): Resultados do *Brasília Heart Study*

**DOI:** 10.36660/abc.20190854

**Published:** 2021-07-15

**Authors:** Joaquim Barreto, Jose Carlos Quinaglia e Silva, Andrei C. Sposito, Luiz Sergio Carvalho

**Affiliations:** 1 Universidade Estadual de CampinasLaboratório de Aterosclerose e Biologia VascularCampinasSPBrasilUniversidade Estadual de Campinas (UNICAMP) - Laboratório de Aterosclerose e Biologia Vascular (Aterolab), Campinas, SP - Brasil; 2 Escola Superior de Ciências da SaúdeBrasíliaDFBrasilEscola Superior de Ciências da Saúde, Brasília, DF - Brasil

**Keywords:** Doenças Cardiovasculares, Fatores de Risco, Mortalidade, Estudos de Coorte, Síndrome Coronariana Aguda, Aterosclerose, Escolaridade

## Abstract

**Fundamento:**

A baixa escolaridade tem sido considerada um fator de risco modificável significativo para o desenvolvimento de doenças cardiovasculares há bastante tempo. Apesar disso, ainda não se sabe muito sobre esse fator impactar ou não os desfechos após infarto do miocárdio com supradesnivelamento do segmento ST (IAMCSST).

**Objetivo:**

Investigar se a escolaridade é um fator de risco independente para mortalidade em pacientes com IAMCSST.

**Métodos:**

Os pacientes com diagnóstico de IAMCSST foram consecutivamente incluídos em uma coorte prospectiva (*Brasília Heart Study*) e categorizados de acordo com os anos dos quartis de estudo (0-3, 4-5, 6-10 e >10 anos). Os grupos foram comparados pelo teste t de Student para variáveis contínuas e qui-quadrado para categóricas. A incidência de mortalidade por todas as causas foi comparada com Kaplan-Meyer com regressão de Cox ajustada por idade, sexo e escore GRACE. Valores de p < 0,05 foram considerados significativos. SPSS21.0 foi utilizado para todas as análises.

**Resultados:**

A média de escolaridade foi de 6,63±4,94 anos. Durante o período de acompanhamento (média: 21 meses; até 6,8 anos), 83 pacientes vieram à óbito (mortalidade cumulativa de 15%). A taxa de mortalidade foi maior entre o quartil inferior em comparação com aqueles do quartil superior [18,5 vs. 6,8%; RR 2,725 (IC 95%: 1,27-5,83; p=0,01)]. Na análise multivariada, a baixa escolaridade perdeu significância estatística para mortalidade por todas as causas após ajuste para idade e sexo, com RR 1,305 (IC 95%: 0,538-3,16; p=0,556), e após ajuste pelo escore GRACE com RR 1,767 (IC 95%: 0,797-3,91; p=0,161).

**Conclusão:**

Investigar se a escolaridade é um fator de risco independente para mortalidade em pacientes com IAMCSST.

## Introdução

Nas últimas décadas, um grande esforço tem sido feito para prevenir fatores de risco modificáveis para doenças cardiovasculares. Entre outros, o baixo nível socioeconômico, avaliado por anos de estudo é destacado como um fator multifacetado que impacta as taxas de incidência e mortalidade de infarto do miocárdio (IM).^1^ Um motivo plausível é o vínculo entre educação e alfabetização em saúde, que compreende a capacidade de reconhecer informações sobre saúde e realizar práticas de autocuidado com eficiência.^2^ De acordo com essa hipótese, aqueles com maior nível de instrução são, provavelmente, mais aderentes às instruções terapêuticas após o evento causador, o que pode, em última instância, favorecer o prognóstico.^3^ Por outro lado, pessoas com menor nível de instrução podem apresentar maior prevalência de comorbidades,^3^ e, com frequência, apresentam acesso tardio aos serviços de saúde,^2^ o que leva ao acesso limitado a estratégias de reperfusão e a um aumento das taxas de mortalidade.

Em cuidados cardiovasculares, a hipótese mencionada é apoiada por um crescente número de evidências, sugerindo que a mortalidade em longo prazo é muito maior entre os pacientes com menor escolaridade. Embora essa relação seja atualmente bem fundamentada, a maioria dos dados foi coletada em países de alta renda, como a Noruega,^4,5^ os Estados Unidos^6^ e a Alemanha.^7^ Nesses países, como resultado de um excelente serviço educacional geral, a escolaridade pode desempenhar um papel mais amplo na alfabetização em saúde do que nos países em desenvolvimento, nos quais a educação continua enfrentando desafios devido à falta de recursos e taxas de abandono implacáveis nos estágios iniciais de escolaridade.^8,9^ Portanto, saber se a escolaridade permanece como um fator de risco modificável significativo para doença cardiovascular em países de baixa e média renda permanece uma questão sem resposta.

Até o momento, resultados anteriores sugerem que aqueles com menor escolaridade têm uma incidência maior de IM no Brasil. No entanto, se a sobrevida global também é determinada por esses fatores, não se sabe ao certo.^10^ Como as doenças das artérias coronárias continuam sendo a principal causa de morte no país, deve-se destacar o papel da escolaridade como um marcador substituto plausível de risco de mortalidade.^11^ Nesse cenário, o presente estudo investigou se a menor escolaridade é um fator de risco independente para mortalidade e estimou seu impacto na saúde cardiovascular em uma coorte brasileira de pacientes com IM.

## Métodos

### População de estudo

Pacientes do *Brasília Heart Study* foram admitidos prospectivamente no estudo (ClinicalTrials.gov Identifier: NCT02062554), um estudo de coorte em andamento cujos detalhes foram publicados em outro local.^12^ Dos 662 pacientes incluídos entre junho de 2006 e novembro de 2016, 542 foram incluídos nesta análise e 120 foram excluídos devido à falta de dados. Resumidamente, o presente estudo envolveu pacientes de qualquer idade, internados por IAM com supradesnivelamento do segmento ST (IAMCSST) em um hospital público de alta complexidade (terciário) (Hospital de Base do Distrito Federal, Brasília, Distrito Federal, Brasil). Os critérios de admissão incluíram: (i) menos de 24 horas do início dos sintomas de IM; (ii) desnivelamento do segmento ST de pelo menos 1 mm (plano frontal) ou 2 mm (horizontal) em derivações contíguas; e (iii) necrose do miocárdio, tal como evidenciado por um aumento de, pelo menos, um valor acima do percentil 99 acima do limite de referência de CQ-MB (25 U/L) e troponina I (0,04 ng/mL), seguido por um declínio de ambos.

Em 24h após admissão hospitalar, amostras de sangue foram coletadas após jejum de 12h e análises bioquímicas foram realizadas para as seguintes medidas: creatina quinase-MB, colesterol total e frações, proteína C reativa (PCR), glicemia de jejum, hemoglobina glicada, creatinina e triglicerídeos. As fórmulas de Cockcroft-Gault e Friedewald foram utilizadas para estimar a depuração e o LDL-c, respectivamente. Todas as análises bioquímicas foram realizadas no mesmo laboratório clínico, certificado pelo Programa de Acreditação de Laboratórios Clínicos da Sociedade Brasileira de Patologia Clínica.

### Definição dos grupos

**Figura 1:**
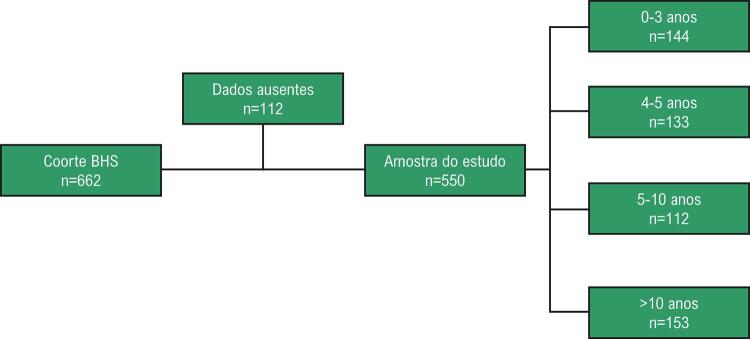
– Fluxograma para os participantes do presente estudo.

Na admissão hospitalar, os pacientes foram interrogados sobre sua escolaridade. O número relatado de anos de estudo foi então registrado quando viável ou presumido de acordo com o nível de escolaridade mais alto atingido pelo paciente. Nesse ponto, os anos de escolaridade considerados segundo o sistema educacional brasileiro, como apresentado a seguir, foram: analfabetos (<4^º^ ano), ensino fundamental (8^º^ ano), ensino médio (11^º^ ano) e educação superior (>15^º^ ano). Finalmente, os participantes foram divididos em anos de quartis de estudo, da seguinte forma: 0-3, 4-5, 6-10 e >10 anos de estudo (Figura 1).

### Acompanhamento e estágios finais

Os pacientes foram acompanhados por meio de consulta ambulatorial mensal ou contato telefônico. O tempo mediano de seguimento foi de 611 (IIQ:724) dias, variando de 1 a 2.504 dias. O desfecho primário do estudo foi a mortalidade por todas as causas. O desfecho composto secundário foram eventos cardíacos adversos maiores (MACE), definidos como IM fatal ou não fatal, morte cardiovascular hospitalar e morte cardíaca súbita. Outros desfechos registrados foram acidente vascular cerebral não fatal, trombose *intra-stent* e angina. Para todos os desfechos, as informações foram obtidas de registros médicos e atestados de óbito.

### Análise estatística

Os dados são média ± desvio padrão para dados normalmente distribuídos, e as variáveis categóricas são apresentadas como porcentagens (%). A normalidade das variáveis quantitativas foi avaliada com o teste de Kolmogorov-Smirnov. As comparações entre 0-3 e >10 grupos de escolaridade foram realizadas com o teste do qui-quadrado para variáveis categóricas e o teste t de Student pareado para variáveis contínuas. As curvas de sobrevivência foram analisadas com o método de Kaplan-Meier e comparadas com o teste Log Rank Mantel-Cox. O modelo de riscos proporcionais de Cox foi adotado para examinar a associação entre escolaridade e tempo para MACE, no qual três modelos predefinidos foram utilizados [modelo 1: não ajustado; modelo 2: ajustado para sexo e idade; modelo 3: ajustado para escore GRACE]. Um valor de p bilateral de <0,05 foi considerado estatisticamente significativo. As análises estatísticas foram realizadas no SPSS para Mac, versão 20.0.

## Resultados

A média de escolaridade foi de 6,63±4,94 anos. As características da linha de base de acordo com os anos do quartil de estudo estão detalhadas na Tabela 1. Um fator de maior importância é que aqueles com menos escolaridade eram mais velhos e mostraram taxas ligeiramente mais baixas de tratamento com estatina após IAMCSST, índice de massa corporal (IMC) mais baixo, além de taxas mais altas de hipertensão. No entanto, as comorbidades [tabagismo, diabetes, dislipidemia], estratégia de reperfusão e atraso no início dos sintomas até a admissão hospitalar foram comparáveis entre os diferentes grupos de escolaridade. A comparação entre os quartis intermediários mostrou que aqueles com seis a 10 anos de escolaridade eram significativamente mais jovens e, em sua maioria, do sexo masculino, IAM prévio e histórico familiar de doença arterial coronariana (DAC) quando comparados a aqueles com quatro ou cinco anos de escolaridade (Tabela 1).

**Tabela 1:** – Características da amostra

	Escolaridade, anos				
	0 - 3	4 - 5	6 - 10	> 10	valor de p^t^
**N**	144	133	112	153	
**Demografia**					
Idade, anos	67,31 ± 12	62,4 ± 12	59,4 ± 11	58,12 ± 10	0,001
Escolaridade, anos	2,4 ± 0,8	4,3 ± 0,5	7,5 ± 0,9	12,8 ± 3,1	0,001
Homem, %	69,4	74,4 ^¶^	84,8	77,1	0,037
IMC, kg/m^2^	26 ± 4,9	26,4 ± 4,3	27,2 ± 4	27,9 ± 4,3	0,003
**Histórico médico**					
MI anterior, %	11,1	9^¶^	16,1	8,5	0,213
Tabagismo, %	37,1	31,6	39,3	37,3	0,613
*Diabetes mellitus*, %	34	29,3	29,5	32,7	0,794
Hipertensão, %	66,7	64,7	61,6	51,6	0,039
Dislipidemia, %	45	47	48	46	0,886
História familiar para DAC, %	33,3	43,6^¶^	57,1	52,3	0,001
**Hemodinâmica**					
PAS, mmHg	132,3 ± 30	133,5 ± 29	138,3 ± 27	139,9 ± 32	0,091
PAD, mmHg	81,9 ± 17	83,8 ± 19	88,5 ± 18	87 ± 21	0,02
Frequência cardíaca, bpm	77,3 ± 18	76,2 ± 17	77,3 ± 16	78,6 ± 16	0,70
GRACE, unidades	150 ± 28	138,5 ± 27	131,9 ± 21	128,7 ± 26	0,001
Fração de ejeção do VE, %	51,5 ± 11	56,8 ± 11	50 ± 10	56 ± 11	0,007
Classificação Killip I, %	83	90	92	92	0,267
**Análises bioquímicas**					
Glicemia de jejum, mg/dL	143,6 ± 65	152 ± 67	150 ± 57	155 ± 77	0,51
Hemoglobina glicosilada, %	6,5 ± 1,7	6,6 ± 1,9	6,3 ± 1,5	6,5 ± 2,1	0,644
CrCl, ml/min/1,73m^2^	65,6 ± 24	72,1 ± 23	71,6 ± 20	71,9 ± 24	0,052
HDL-C, mg/dL	40,2 ± 11,1	37 ± 9,9	37,3 ± 10,5	37,2 ± 11	0,045
LDL-C, mg/dL	125 ± 151	124,6 ± 66	119,1 ± 40	128,3 ± 44	0,894
TG, mg/dL	137,3 ± 88	154,4 ± 113	183,7 ± 148	208,8 ± 258	0,002
CRP, mg/L	1,6 ± 2,9	1,1 ± 1,5	1,4 ± 2,6	1,4 ± 2,5	0,390
Pico de CQ-MB, mg/dL	264 ± 213	290,9 ± 206	281 ± 193	240 ± 169	0,147
Massa do infarto (RMC), g	17,5 ± 9	15,9 ± 9	19,5 ± 13	14 ± 10	0,192
**Tratamento**					
Tenecteplase, %	60,4	63,2	65,2	62,7	0,89
ICP primária, %	50	42,9	50	55,6	0,204
Tempo para reperfusão, min	160,9 ± 149,3	199,8 ± 194	167,5 ± 167	156,7 ± 164	0,139
Tempo até hospital, min	128 ± 111	134 ± 144	130 ± 129	119 ± 127	0,344
Sinvastatina, %	60,1	65,4	76,9	70,7	0,032
**Resultados**					
Mortalidade por todas as causas, n (%)	20 (18,5)	22 (17,3)	16 (9,5)	10 (6,8)	0,016
MACE, n (%)	18 (16,7)	20 (15,7)	22 (13,1)	15 (10,2)	0,515

*US$ 1 = R$ 3,91. ^*t*^Valor de p para comparação entre o quartil mais baixo e o mais alto de escolaridade para variáveis categóricas e contínuas por qui quadrado e teste t de Student pareado, respectivamente. ^*¶*^P<0,05 para o segundo (4-5) vs. terceiro (6-10 anos) quartis. IMC: índice de massa corporal; MI: infarto do miocárdio; DAC: doença arterial coronariana; PAS: pressão arterial sistólica; PAD: pressão arterial diastólica; TG: triglicerídeos; CrCl: depuração da creatinina; CRP: Proteína C-reativa; CQ-MB: creatina quinase M classe B; RMC: ressonância magnética cardíaca; VE: ventricular esquerdo; ICP: intervenção coronária percutânea; MACE: principais eventos cardiovasculares adversos.

**Figura 2:**
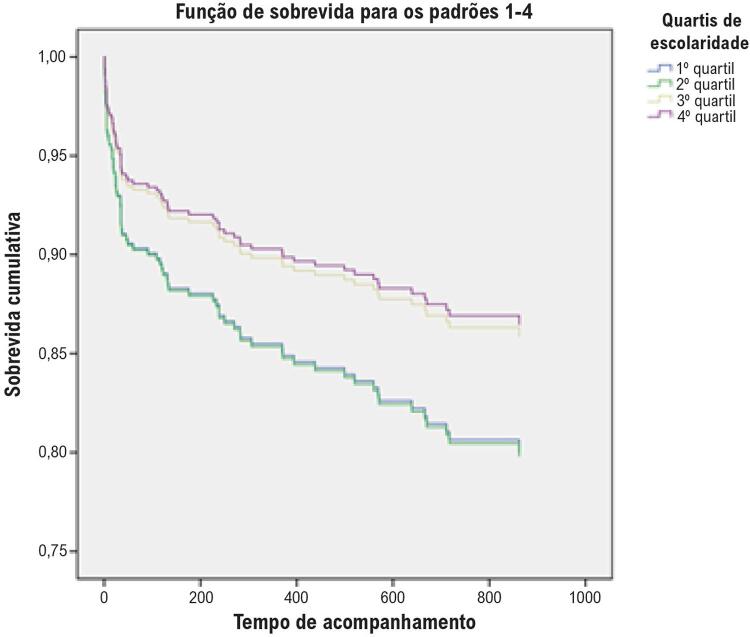
– Kaplan-Meyer para mortalidade por todas as causas estratificada por anos de quartis de estudo.

Durante o período de acompanhamento (média: 21 meses; faixa: 0-6,8 anos), 83 pacientes faleceram (mortalidade cumulativa de 15%). No modelo linear, a escolaridade reduziu significativamente a chance de óbito, de acordo com nosso período de acompanhamento, com RR 0,927 (IC 95: 0,877-0,981; p=0,008). A taxa de mortalidade foi maior entre o quartil inferior em comparação com aqueles com >10 anos de estudo (18,5 vs 6,8%, p=0,016) (Figura 2). Na análise univariada, as seguintes variáveis estiveram relacionadas às maiores taxas de mortalidade: idade (p=0,001), tabagismo (p=0,046), classificação Killip (p=0,013) e escolaridade (p=0,021). Em comparação com indivíduos com >10 anos de escolaridade, ter <3 anos de estudo foi relacionado à mortalidade por todas as causas com RR 2,725 (IC 95%: 1,27-5,83; p=0,01). Na análise multivariada, apenas idade e Killip >I permaneceram significativamente associados à mortalidade. Na comparação dos grupos, ter menos de três anos de estudo perdeu significância estatística após ajuste por idade e sexo, com RR 1,305 (IC 95%: 0,538-3,16; p=0,556) e após ajuste pelo escore GRACE com RR 1,767 (IC 95%: 0,797-3,91; p=0,161) (Tabela 2). Da mesma forma, nenhum dos quartis intermediários foi significativamente relacionado aos resultados na análise multivariada.

**Tabela 2:** – Regressão de Cox para mortalidade por todas as causas

Variável	Modelo 1 (bruto)			Modelo 2^a^			Modelo 3^b^		
	RR	IC 95%	Valor de p	RR	IC 95%	Valor de p	RR	IC 95%	Valor de p
Idade	1,081	1,06-1,11	0,001	1,071	1,05-1,10	0,001	1,079	1,05-1,11	0,001
Homem, %	1,063	0,65-1,75	0,810	1,054	0,61-1,85	0,854	1,012	0,575-1,78	0,967
Dislipidemia	1,218	0,75-1,99	0,428	1,314	0,756-2,28	0,333	1,356	0,779-2,36	0,282
Diabetes mellitus	1,146	0,67-1,96	0,620	0,957	0,531-1,72	0,957	1,055	0,581-1,91	0,860
Hipertensão	0,683	0,42-1,10	0,117	0,723	0,412-1,27	0,258	0,825	0,485-1,40	0,479
Tabagismo	1,685	1,01-2,81	0,046	1,102	0,611-1,99	0,747	1,068	0,607-1,87	0,686
Classificação Killip > I	1,912	1,15-3,18	0,013	1,932	1,13-3,31	0,017	1,985	1,12-3,50	0,018
Quartis de escolaridade			0,021			0,844			0,223
1^o^ vs. 4^o^	2,725	1,27-5,83	0,010	1,305	0,538-3,16	0,556	1,767	0,797-3,91	0,161
2^o^ vs. 4^o^	2,469	1,17-5,22	0,018	1,470	0,634-3,41	0,369	2,206	1,01-4,83	0,048
3^o^ vs. 4^o^	1,386	0,63-3,05	0,419	1,324	0,579-3,024	0,506	1,018	1,01-1,03	0,497

## Discussão

No presente estudo, a baixa escolaridade não teve relação independente com a mortalidade após IAMCSST. Apesar disso, uma taxa de mortalidade 2,7 vezes maior foi encontrada para aqueles com menor escolaridade em comparação ao quartil mais alto nos modelos brutos, resultado que foi neutralizado após o ajuste por idade ou escore GRACE. São muitas as razões possíveis para esse achado.

Não se pode deixar de considerar que os pacientes menos instruídos também eram significativamente mais velhos. Essa discrepância é recorrente em outros estudos, uma vez que um aumento geral nos anos de escolaridade esperados ocorreu globalmente nas últimas décadas. Aliás, relatórios do Programa das Nações Unidas para o Desenvolvimento (PNUD) estimam que os anos de escolaridade esperados aumentaram de 13 para 16 anos em países de alta renda, e de cinco para nove anos em países de baixa e média renda nos últimos 30 anos.^13^ No Brasil, no mesmo período, a média de anos de escolaridade passou de 2,6 para 7,8 anos, diminuindo as taxas de analfabetismo dos últimos 25% para os atuais 9,6%.^13,14^ Visto que a idade é um fator de risco bem estabelecido para mortalidade por qualquer causa, sua relação com a escolaridade prejudica as tentativas de responder se a escolaridade é um marcador substituto independente de mortalidade em pacientes infartados. Portanto, resultados conflitantes são encontrados na literatura (Tabela 3).

**Tabela 3:** – Comparação com outros estudos

Estudo	Dados	Grupos	Resultados	Resultados
Este estudo	n=542, coorte prospectiva de pacientes com IAMCSST, Brasil	<3, 4-6, 7-10, >10 anos de estudo	Mortalidade por todas as causas	Não relacionado independentemente
Mehta et al., 2011	11.326 IAMCSST, retrospectivo, nove países desenvolvidos	< 8 vs. > 16 anos de estudo	Taxa de mortalidade de um ano	cinco vezes maior naqueles pacientes com menor nível de escolaridade; significativo após ajuste para características de linha de base e país em que foi realizado
Strand, Tverdal, 2004^4^	n=44.684, prospectivo, Noruega	Escolaridade alta (nível médio/universidade/faculdade) vs. baixa (ensino fundamental ou sem escolaridade)	Mortalidade por IDH	O ajuste para fatores de risco reduziu o excesso de mortalidade por IDH nos grupos de baixa escolaridade em 91% para homens e 67% para mulheres.
J. Igland et al., 2014^5^	N=111.993; IAM; Noruega	Educação básica vs. educação superior	Mortalidade de 28 dias e um ano	Aumento de 1,18 vezes em um ano e aumento de 1,04 vezes na taxa de mortalidade em 30 dias, para modelo totalmente ajustado para pacientes com idade entre 70-94 anos. Renda incluída no modelo de ajuste.
L. Consuegra-Sanchez et al., 2011^20^	N=5.797; IAM; seguimento prospectivo de oito anos; Espanha;	Sem escolaridade ou educação básica vs. ensino médio ou ensino superior	Mortalidade por todas as causas	Risco 15% maior no grupo de menos escolaridade
Coady et al., 2014^6^	N=15.972; Estados Unidos	Educação inferior ao ensino médio vs. diploma universitário	Mortalidade de longo prazo (1-5 anos)	Aumento de 1,6 e 1,37 vezes na mortalidade a longo prazo para homens e mulheres com menor escolaridade, respectivamente.
Kirchberger et al., 2014^7^	N=3.419; IAM; Alemanha	Escolaridade baixa (sem treinamento vocacional formal completo) vs. escolaridade alta	Mortalidade no longo prazo	Nenhum efeito da educação sobre a mortalidade foi encontrado para a amostra total. Em pacientes com mais de 65 anos, aqueles com menor escolaridade tiveram taxa de mortalidade 1,4 vezes maior.

Dessa forma, Kirchberger et al.,^7^ analisaram dados de 3.400 pacientes com IAM, que foram agrupados com níveis de escolaridade baixa ou alta, usando um corte de 13 anos de escolaridade. De acordo com nossos achados, embora a baixa escolaridade estivesse relacionada a um aumento de 1,46 vezes na taxa de mortalidade na análise bruta, o ajuste por idade a tornou estatisticamente não significativa.^7^ Além disso, de acordo com os motivos anteriormente expostos, a escolaridade voltou a ter significância estatística quando os pacientes foram estratificados por faixas etárias.^7^

Em contraste, Mehta et al.,^15^ relataram um aumento de cinco vezes na mortalidade entre os pacientes com menos escolaridade e IAMCSST, que permaneceu significativa após o ajuste por idade.^15^ Além disso, ao passo que o grupo de menor escolaridade compreendia 2.249 indivíduos, aqueles com >16 anos de escolaridade foram somente 469 pacientes.^15^ Essa diferença pode ter minado a importância do efeito das disparidades de idade nos resultados.^15^ O estudo também analisou dados coletados em nove países de alta renda.^15^ Portanto, os altos padrões de educação desses países podem ter impulsionado sua contribuição para os resultados de saúde a um nível suficiente para não ser excedido pelo efeito das discrepâncias de idade. Nesse sentido, Mehta et al.^15^ incluíram dados incluídos da Noruega, atualmente o 1^º^ país no *ranking *de educação, ao passo que o Brasil se destaca como o 87^º^ país em termos de educação, portanto fornecendo explicações razoáveis para as discrepâncias relatadas.^13,14^ Ademais, Mehta et al.,^15^ compararam seus grupos com uma escolaridade superior (>16 anos) do que o que foi realizado em nosso estudo (>10 anos), o que pode ter alimentado significativamente o tamanho do efeito verificado.

Finalmente, é importante destacar que a renda é, de longe, o fator mais estreitamente relacionado aos resultados clínicos após IAMCSST entre os fatores socioeconômicos.^16,17^ Nesse sentido, pode-se argumentar que o impacto da educação nos resultados clínicos resultaria em parte de sua relação com a renda, que é plausivelmente maior entre os países de alta renda, onde a riqueza é distribuída de forma mais justa. Aliás, a renda média anual dos brasileiros sem nenhum nível de instrução é de US$ 3.070,^18^ cerca de 85% menor do que a renda daqueles com o mesmo nível de instrução nos Estados Unidos (US$ 20 mil).^19,20^ Da mesma forma, a escolaridade foi apenas ligeiramente relacionada à renda (R=0,3) no presente estudo, o que se traduz plausivelmente em um impacto mais leve da escolaridade no acesso aos serviços de saúde e melhora geral dos resultados clínicos, fornecendo um mecanismo viável para as discrepâncias relatadas.

### Limitações e pontos fortes do estudo

O presente estudo apresenta diversas limitações. Em primeiro lugar, o número de pacientes era menor do que em estudos anteriores. Em segundo lugar, nossos grupos tiveram prevalências divergentes de fatores de risco conhecidos, principalmente a idade. A coorte não incluiu pacientes com um suposto novo bloqueio de ramo esquerdo como IAMCSST na admissão hospitalar. Por fim, embora seja validada e amplamente utilizada, a divisão em grupos por anos de escolaridade subestima o papel do conteúdo sobre a quantidade de anos estudados, o que pode adicionar um viés indesejável à nossa análise, conforme discutido anteriormente.

Por outro lado, há diversos pontos fortes no presente estudo. Mais importante ainda, é um dos poucos estudos a avaliar prospectivamente o impacto da escolaridade nos resultados de IAMCSST em um país em desenvolvimento. Além disso, o estudo reforçou que os resultados obtidos em países de alto desenvolvimento não podem ser extrapolados para o cenário brasileiro.

## Conclusão

A baixa escolaridade não foi um preditor independente de morte nem MACE após IAMCSST no presente estudo.
