# Supplementary material for: O Impacto da Educação na Mortalidade por Todas as Causas após Infarto do Miocárdio com Supradesnivelamento do Segmento ST (IAMCSST): Resultados do Brasília Heart Study
Source: Arq Bras Cardiol. 2021 Jul 15;117(1):5–12. [Article in Portuguese] doi: 10.36660/abc.20190854 (PMC8294733; doi:10.36660/abc.20190854)
Supplement: Translation

# Translation: The Impact of Education on All-cause Mortality Following St-Segment Elevation Myocardial Infarction (STEMI): Results from the Brazilian Heart Study

**Authors:** Joaquim Barreto, Jose Carlos Quinaglia e Silva, Andrei C. Sposito, Luiz Sergio Carvalho

**Affiliations:** 1 BrasilUniversidade Estadual de Campinas (UNICAMP) - Laboratório de Aterosclerose e Biologia Vascular (Aterolab), Campinas, SP - Brasil; 2 BrasilEscola Superior de Ciências da Saúde, Brasília, DF - Brasil

**Keywords:** Cardiovascular Diseases, Risk Factors, Mortality, Cohort Studies, Acute Coronary Syndrome, Atherosclerosis, Scholarity

## Abstract

**Background:**

Low schooling has been considered an important modifiable risk factor for the development of cardiovascular disease for a long time. Despite that, whether this factor impacts the outcomes following ST-segment elevation myocardial infarction (STEMI) is poorly understood.

**Objective:**

To investigate whether schooling stands as an independent risk factor for mortality in STEMI patients.

**Methods:**

STEMI-diagnosed patients were consecutively enrolled from a prospective cohort (Brasilia Heart Study) and categorized according to years of study quartiles (0-3, 4-5, 6-10 and >10 years). Groups were compared by student’s t test for continuous variables and qui-square for categorical. Incidence of all-cause mortality was compared with Kaplan-Meyer with Cox regression adjusted by age, gender, and GRACE score. Values of p < 0.05 were considered significant. SPSS21.0 was used for all analysis.

**Results:**

The mean schooling duration was 6.63±4.94 years. During the follow-up period (mean: 21 months; up to 6.8 years), 83 patients died (cumulative mortality of 15%). Mortality rate was higher among the lowest quartile compared to those in the highest quartile [18.5 vs 6.8%; HR 2.725 (95% CI: 1.27-5.83; p=0.01)]. In multivariate analysis, low schooling has lost statistical significance for all-cause mortality after adjustment for age and gender, with HR of 1.305 (95% CI: 0.538-3.16; p=0.556), and after adjustment by GRACE score with an HR of 1.767 (95% CI: .797-3.91; p=0.161).

**Conclusion:**

Low schooling was not an independent risk factor for mortality in STEMI patients.

## Introduction

Over the past decades, a great effort has been spared towards the prevention of modifiable risk factors for cardiovascular disease. Among others, low socioeconomic status, assessed by years of study, stands as a multifaceted factor that impacts both incidence and mortality rates of myocardial infarction (MI).^1^ One plausible reason is the link between education and health literacy, which comprises the capacity to acknowledge health information and efficiently perform self-care practices.^2^ According to this hypothesis, those with higher level of instruction are more likely adherent to therapeutical instructions following the index event, which may ultimately favor prognosis.^3^ On the other hand, those least instructed may present a higher prevalence of comorbidities^3^ and frequently show delayed access to healthcare facilities,^2^ leading to limited access to reperfusion strategies and increased rates of death.

In cardiovascular care, the aforementioned hypothesis is supported by a growing body of evidence, suggesting that long-term mortality among the least educated patients is greatly increased. Though such link is now well-supported, most data were collected from high-income nations, such as Norway,^4,5^ the United States,^6^ and Germany.^7^ In these countries, as a result of an overall excellent educational service, schooling may play a wider role on health literacy than it does in developing countries, in which education remains challenged by lack of resources and relentless drop-out rates in the earlier stages of instruction.^8,9^ Therefore, whether schooling stands as a significant modifiable risk factor for cardiovascular disease in low-to-middle income countries remains unanswered.

To date, previous results suggest that those least educated have a higher incidence of MI in Brazil. Nonetheless, whether overall survival is also determined by this factors remains unknown.^10^ As coronary artery diseases remain the main cause of death in this country, elucidating the role of schooling as a plausible surrogate marker of mortality risk is mandatory.^11^ In this scenario, the present study investigated if lower schooling stands as an independent risk factor for mortality and estimated its impact on cardiovascular health in a Brazilian cohort of MI patients.

## Methods

### Study population

We prospectively enrolled patients from the Brasília Heart Study (ClinicalTrials.gov Identifier: NCT02062554), an ongoing cohort study of which details are published elsewhere.^12^ Out of 662 consecutive patients included between June/2006 and November/2016, 542 were included in this analysis and 120 were excluded due to missing data. Briefly, this study enrolled patients of any age admitted for ST-segment elevation MI (STEMI) at a public high-complexity (tertiary) hospital (Hospital de Base do Distrito Federal, Brasília City, Federal District, Brazil). The admission criteria included: (i) less than 24 hours from the onset of symptoms of MI; (ii) ST-segment elevation of at least 1 mm (frontal plane) or 2 mm (horizontal) in contiguous leads; and (iii) myocardial necrosis, as evidenced by an increase to at least one value above the 99^th^ percentile above the reference limit of CK-MB (25 U/L) and troponin I (0.04 ng/mL), followed by a decline of both.

Within 24h of hospital admission, blood samples were collected after 12h fasting and biochemical analysis was performed for the following measurements: creatine kinase-MB, total cholesterol and fractions, C-reactive protein, fasting glucose, glycated haemoglobin, creatinine, and triglycerides. Cockcroft-Gault and Friedewald formulas were used to estimate clearance and LDL-c, respectively. All biochemical analyses were performed in the same clinical laboratory certified by the Accreditation Program of Clinical Laboratories of the Brazilian Society of Clinical Pathology.

### Groups Definition

At hospital admission, patients were asked about their schooling. The informed number of schooling years was then registered when feasible or presumed according to the highest level of education achieved by the patient. In this matter, schooling years according to the Brazilian educational system, as follows, were considered: illiterate (<4^th^ year), primary education (8^th^ year), high school (11^th^ year), and college education (>15^th^ year). Finally, subjects were divided in years of study quartiles, as follows: 0-3, 4-5, 6-10, and >10 years of study (Figure 1).

**Figure 1:** – Flowchart for participants in the present study.

### Follow up and endpoints

Patients were followed with monthly outpatient clinic visit or telephone contact. The median follow-up time was of 611 (IQR:724) days, ranging from 1 to 2,504 days. The primary endpoint of the study was all-cause mortality. The secondary composite outcome was major adverse cardiac events (MACE), defined as fatal or non-fatal MI, in-hospital cardiovascular death and sudden cardiac death. Other outcomes registered comprised: non-fatal stroke, intra-stent thrombosis, and angina. For all endpoints, information was obtained from medical records and death certificates.

### Statistical analysis

Data are mean ± standard deviation for normally distributed data, and categorical variable are presented as percentages (%). The normality of the quantitative variables was assessed with the Kolmogorov-Smirnov test. Comparisons between 0-3 and >10 schooling groups were performed using the chi-square test for categorical variables, and paired student’s t-test, for continuous variables. Survival curves were analyzed with the Kaplan-Meier method and compared with the Log Rank Mantel-Cox test. Cox proportional hazards model was used to examine the association between schooling and time to MACE, in which three pre-defined models were used [model 1: unadjusted; model 2: adjusted for sex, age; model 3: adjusted for GRACE score]. A two-sided p-value of < 0.05 was considered statistically significant. Statistical analyses were performed using SPSS for Mac, version 20.0.

## Results

The mean schooling duration was 6.63±4.94 years. Baseline characteristics according to years of study quartile are detailed in Table 1. Most importantly, those least educated were older and showed slightly lower rates of statin treatment following STEMI, lower body mass index (BMI) as well as higher rates of hypertension. However, comorbidities [smoking, diabetes, dyslipidaemia], reperfusion strategy, and delay from symptoms onset to hospital admission were all comparable across schooling groups. Comparison between intermediate quartiles showed that those with 6-10 years of schooling were significantly younger and were mostly male, prior MI, and family history of coronary artery disease (CAD) when compared to those with 4-5 years of schooling (Table 1).

**Table 1:** – Sample characteristics

	Schooling, years				
	0 - 3	4 - 5	6 - 10	>10	p-value^t^
**N**	**144**	**133**	**112**	**153**	
**Demographics**					
Age, years	67.31 ± 12	62.4 ± 12^¶^	59.4 ± 11	58.12 ± 10	.001
Schooling, years	2.4 ± 0.8	4.3 ± 0.5^¶^	7.5 ± 0.9	12.8 ± 3.1	.001
Male, %	69.4	74.4^¶^	84.8	77.1	.037
BMI, kg/m^2^	26 ± 4.9	26.4 ± 4.3	27.2 ± 4	27.9 ± 4.3	.003
**Medical History**					
Prior MI, %	11.1	9^¶^	16.1	8.5	.213
Smoking, %	37.1	31.6	39.3	37.3	.613
*Diabetes mellitus*, %	34	29.3	29.5	32.7	.794
Hypertension, %	66.7	64.7	61.6	51.6	.039
Dyslipidemia, %	45	47	48	46	.886
Family history for CAD, %	33.3	43.6^¶^	57.1	52.3	.001
**Hemodynamics**					
SBP, mmHg	132.3 ± 30	133.5 ± 29	138.3 ± 27	139.9 ± 32	0.091
DBP, mmHg	81.9 ± 17	83.8 ± 19	88.5 ± 18	87 ± 21	.02
Heart Rate, bpm	77.3 ± 18	76.2 ± 17	77.3 ± 16	78.6 ± 16	0.70
GRACE, units	150 ± 28	138.5 ± 27	131.9 ± 21	128.7 ± 26	.001
LV Ejection fraction, %	51.5 ± 11	56.8 ± 11	50 ± 10	56 ± 11	.007
Killip class I, %	83	90	92	92	.267
**Biochemical Analyses**					
Fasting glucose, mg/dL	143.6 ± 65	152 ± 67	150 ± 57	155 ± 77	.51
Glycosylated hemoglobin, %	6.5 ± 1.7	6.6 ± 1.9	6.3 ± 1.5	6.5 ± 2.1	.644
CrCl, ml/min/1.73m^2^	65.6 ± 24	72.1 ± 23	71.6 ± 20	71.9 ± 24	.052
HDL-C, mg/dL	40.2 ± 11.1	37 ± 9.9^*¶*^	37.3 ± 10.5	37.2 ± 11	.045
LDL-C, mg/dL	125 ± 151	124.6 ± 66	119.1 ± 40	128.3 ± 44	.894
TG, mg/dL	137.3 ± 88	154.4 ± 113	183.7 ± 148	208.8 ± 258	.002
CRP, mg/L	1.6 ± 2.9	1.1 ± 1.5	1.4 ± 2.6	1.4 ± 2.5	.390
CK-MB peak, mg/dL	264 ± 213	290.9 ± 206	281 ± 193	240 ± 169	.147
Infarction mass (CMR), g	17.5 ± 9	15.9 ± 9	19.5 ± 13	14 ± 10	.192
**Treatment**					
Tenecteplase, %	60.4	63.2	65.2	62.7	.89
Primary PCI, %	50	42.9	50	55.6	.204
Time to reperfusion, min	160.9 ± 149.3	199.8 ± 194	167.5 ± 167	156.7 ± 164	.139
Time to hospital, min	128 ± 111	134 ± 144	130 ± 129	119 ± 127	.344
Simvastatin, %	60.1	65.4	76.9	70.7	.032
**Outcomes**					
All-cause death, n (%)	20 (18.5)	22 (17.3)^ ¶^	16 (9.5)	10 (6.8)	.016
MACE, n (%)	18 (16.7)	20 (15.7)	22 (13.1)	15 (10.2)	.515

*US$ 1 = R$ 3.91. ^t^P-value for comparison between lowest and highest quartile of schooling for categorical and continuous variables by qui square and paired student’s t-test, respectively. ^¶^P<0.05 for second (4-5) vs. third (6-10 years) quartiles. BMI: body mass index; MI: myocardial infarction; CAD: coronary artery disease; SBP: systolic blood pressure; DBP: diastolic blood pressure; TG: triglycerides; CrCl: creatinine clearance; CRP: C-reactive protein; CK-MB: creatine kinase M class B; CMR: cardiac magnetic resonance; LV: left ventricular; PCI: percutaneous coronary intervention; MACE: major adverse cardiovascular events.

During the follow-up period (mean: 21 months; range: 0-6.8 years), 83 patients died (cumulative mortality of 15%). In linear model, schooling significantly reduced the chance of dying, according to our follow up period, with HR of 0.927 (95CI: 0.877-0.981; p=0.008). Mortality rate was higher among the lowest quartile compared to those with >10 years of study (18.5 vs 6.8%, p=0.016) (Figure 2). In univariate analysis, the following variables were related to higher mortality rates: age (p=0.001), smoking (p=0.046), Killip class (p=0.013), and schooling (p=0.021). Compared to individuals with >10 years of schooling, having <three years of study was related to all-cause mortality with an HR of 2.725 (95% CI: 1.27-5.83; p=0.01). In multivariate analysis, only age and Killip > I remained significantly associated to mortality. In group comparison, having less than three years of study has lost statistical significance after adjustment by age and gender, with HR of 1.305 (95% CI: 0.538-3.16; p=0.556), and after adjustment by GRACE score with HR of 1.767 (95% CI: .797-3.91; p=161) (Table 2). Similarly, neither of intermediate quartiles were significantly related to outcomes in multivariate analysis.

**Figure 2:** – Kaplan-Meyer of all-cause mortality stratified by years of study quartiles

**Table 2:** – Cox regression of all-cause mortality

Variable	Model 1 (Crude)			Model 2^a^			Model 3^b^		
	HR	95%CI	p*-value*	HR	95%CI	p*-value*	HR	95%CI	p*-value*
Age	1.081	1.06-1.11	.001	1.071	1.05-1.10	.001	1.079	1.05-1.11	.001
Male	1.063	0.65-1.75	.810	1.054	0.61-1.85	.854	1.012	.575-1.78	.967
Dyslipidemia	1.218	0.75-1.99	.428	1.314	.756-2.28	.333	1.356	.779-2.36	.282
Diabetes mellitus	1.146	0.67-1.96	.620	.957	.531-1.72	.957	1.055	.581-1.91	.860
Hypertension	0.683	0.42-1.10	.117	.723	.412-1.27	.258	.825	.485-1.40	.479
Smoking	1.685	1.01-2.81	.046	1.102	.611-1.99	.747	1.068	.607-1.87	.686
Killip class > I	1.912	1.15-3.18	.013	1.932	1.13-3.31	.017	1.985	1.12-3.50	.018
Schooling Quartiles			.021			.844			.223
1^st^ vs. 4^th^	2.725	1.27-5.83	.010	1.305	.538-3.16	.556	1.767	.797-3.91	.161
2^nd^ vs. 4^th^	2.469	1.17-5.22	.018	1.470	.634-3.41	.369	2.206	1.01-4.83	.048
3^rd^ vs. 4^th^	1.386	0.63-3.05	.419	1.324	.579-3.024	.506	1.018	1.01-1.03	.497

## Discussion

In the present study, low schooling was not independently related to mortality after STEMI. Despite that, a 2.7-fold increased mortality rate was found for those least educated in comparison to the highest quartile in crude models, result which was neutralized after adjustment by age or GRACE score. Many are the possible reasons for this finding.

It may not be unconsidered that least educated patients were also significantly older. Such discrepancy is recurrent in other studies, as an overall increase in expected schooling years has occurred globally over the past decades. In fact, United Nations Development Programme (UNDP) reports estimate that the expected schooling years rose from 13 to 16 years in high-income countries, and from five to nine years in low and middle-income countries over the past 30 years.^13^ In Brazil, in the same period, the mean schooling years rose from 2.6 to 7.8 years, decreasing illiteracy rates from past 25% to current 9.6%.^13,14^ As age stands as a well-stablished risk factor for mortality from any cause, its link to schooling undermines the attempts to answer whether schooling is an independent surrogate marker of mortality in infarcted patients. Therefore, conflicting results are found in the literature (Table 3).

**Table 3:** – Comparison with other studies

Study	Data	Groups	Outcomes	Results
Present study	n=542, prospective cohort of STEMI patients, Brazil	<3, 4-6, 7-10, >10 years of study	All-cause mortality	Not independently related
Mehta et al., 2011	11,326 STEMI, retrospective, 9 developed countries	< 8 vs. > 16 years of study	One-year mortality rate	five-fold higher in those least educated patients; significant after adjustment for baseline characteristics and country of enrollment
Strand, Tverdal, 2004^4^	n=44,684, prospective, Norway	High education (secondary level/ university/ college) vs. low (primary or no schooling)	IHD mortality	Adjustment for risk factors reduced excess IHD mortality in the low educational groups by 91% for men and 67% for women.
J. Igland et al., 2014^5^	N=111,993; AMI; Norway	Basic vs. tertiary education	28-day and one-year mortality	1.18-fold increase in one year, and 1.04-fold increase in 30-day mortality rate, for fully adjusted model for patients aged 70-94 years old. Included income in adjustment model.
L. Consuegra-Sanchez et al., 2011^20^	N=5797; AMI; eight years prospective follow-up; Spain;	No schooling or basic education vs. Secondary school or higher education	All-cause mortality	15% higher risk in the least educated group
Coady et al., 2014^6^	N=15972; United States	Lower than high school education vs. college degree	Long-term mortality (1-5 years)	1.6- and 1.37-fold increased long-term mortality for the least educated men and women, respectively.
Kirchberger et al., 2014^7^	N=3419; AMI; Germany	Low education (without completed formal vocational training) vs. High education	Long-term mortality	No effect of education on mortality was found for the total sample. In 65+ years old patients, those least educated had 1.4-fold increased mortality rate.

Accordingly, Kirchberger et al.^7^ analyzed data from 3,400 MI patients, which were grouped as low or high educational backgrounds using a cut-off of 13 schooling years. In accordance to our findings, though low schooling was related to a 1.46-fold increased mortality rate in crude analysis, adjustment by age has rendered it statistically not significant.^7^ Furthermore, in agreement with the previously exposed reasons, schooling regained statistical significance when patients were re-stratified in age groups.^7^

Contrastingly, Mehta et al.^15^ reported a five-fold increased mortality among least educated STEMI patients, which remained significant after adjustment by age.^15^ Of note, whereas least educated group comprised 2,249 subjects, those with >16 years of study were only 469 patients.^15^ Such difference may have undermined the effect size of age disparities on outcomes.^15^ Besides that, the study analyzed data collected from nine high-income countries.^15^ Therefore, the high standards of education delivered in these countries may have powered its contribution to health outcomes to a sufficient level for it not to be exceeded by the effect of age discrepancies. In this matter, Mehta et al.^15^ included data from Norway, currently the 1^st^ in educational ranking, whereas Brazil stands as the 87^th^ country in terms of education, hence providing reasonable explanations for the discrepancies reported.^13,14^ Moreover, Mehta et al.^15^ compared its groups to a higher schooling (>16 years) then our study did (>10 years), which may have significantly fuelled the verified effect size.

Finally, highlighting that income is by far the most narrowly related to clinical outcomes following STEMI among socioeconomic factors is important.^16,17^ In this sense, one may argue that the impact of education on clinical outcomes would in part result from its relation to income, which is plausibly higher among high-income countries, where wealthiness is more fairly distributed. In fact, the median annual income of Brazilian citizens with no instruction level is US$3,070,^18^ nearly 85% lower than the income for those with the same instruction level in the United States (US$20,000).^19^ Correspondingly, schooling was only weakly related to income (R=0.3) in the present study, which plausibly translates into a slighter impact of schooling on access to health care services and overall improvement of clinical outcomes, providing a plausible mechanism for the reported discrepancies.

### Study limitations and strengths

The present study has several limitations. Firstly, the number of patients was lower than in previous studies. Secondly, our groups had divergent prevalence of known risk factors, chiefly age. The cohort did not include patients with presumably new left branch block as a STEMI at hospital admission. Finally, though it is validated and widely used, the division in groups according to schooling years underestimate the role of content over quantity of years studied, which may add an undesired bias to our analysis, as previously discussed.

On the other hand, many are the strengths of this study. Most importantly, it stands as one of the few studies to prospectively evaluate the impact of schooling on STEMI outcomes in a developing country. Furthermore, it reinforced that results obtained from high-development countries may not be extrapolated to the Brazilian scenario.

## Conclusion

Low schooling was not an independent predictor of death nor MACE following STEMI in the present study.
